# Diagnosing Lung Cancers through Examination of Micro-RNA Biomarkers in Blood, Plasma, Serum and Sputum: A Review and Summary of Current Literature

**DOI:** 10.3390/ijms17040494

**Published:** 2016-04-01

**Authors:** Jennifer Gyoba, Shubham Shan, Wilson Roa, Eric L. R. Bédard

**Affiliations:** 1 Division of Thoracic Surgery, University of Alberta, Edmonton, AB T6G 2R3, Canada; sshan1@ualberta.ca; 2 Department of Oncology, University of Alberta, Edmonton, AB T6G 2R3, Canada; wilson.roa@albertahealthservices.ca

**Keywords:** lung cancer, micro-RNA, whole blood, serum, plasma, sputum, early detection, screening, biomarker

## Abstract

Lung cancer is the leading cause of cancer related morbidity and mortality worldwide. Currently, the vast majority of lung cancers are diagnosed at a late stage, when patients become symptomatic leading to dismal, less than 15% five-year survival rates. Evidence has demonstrated that screening computed tomography scans can be used to detect lung cancer, but these scans have high false positive rates. Therefore, there is a continued need for the development of minimally-invasive methods to screen the high risk population and diagnose lung cancer at an earlier, curable stage. One such promising area is the use micro-RNAs. These are short, non-coding RNA molecules that have been shown in previous research to be dysregulated in cancers. This review will focus on the potential use of miRNA levels in various biological fluids (whole blood, plasma, serum, and sputum) and demonstrate their potential utility as screening and diagnostic biomarkers for lung cancer. Current research will be analyzed and compared, and future directions in establishing the use of miRNAs for detecting lung cancer will be discussed.

## 1. Lung Cancer Statistics

Lung cancer is the leading cause of morbidity and mortality associated with malignancies worldwide, with rates increasing in the past decade due to aging, pollution, and increased voluntary or involuntary consumption of various carcinogens [1,2,3]. During the early stages, patients with lung cancer are largely asymptomatic which results in the high rate of diagnosis when curative treatments are mostly ineffective. In fact, approximately 75% of lung cancers are diagnosed at locally advanced or metastatic stages (stage III/IV) resulting in a less than 15% 5-year survival rate [2,4,5].

Pathologically, lung cancers are divided into two main categories, non-small cell lung cancers (NSCLC) and small-cell lung carcinoma (SCLC). NSCLC accounts for over 80% of the lung cancers and is classified into the subtypes adenocarcinoma (AC), squamous-cell carcinoma (SCC), and large cell carcinoma (LCC) [1,4,5]. The 5-year survival rate for a patient with stage 1A NSCLC is 80% and this drops dramatically to under 10% for those with stage IV NSCLC. Given the high prevalence of NSCLC and low survival rates, efforts have been focused on developing screening protocols for the high risk population likely to develop NSCLC. A cheap and reproducible test with the sensitivity of a screening test and the specificity of a diagnostic test has not yet been achieved. Development of such a test would assist patients in detecting their cancer earlier so they could receive treatment as soon as possible and increase their survival rates.

## 2. Current Screening and Diagnostics for Lung Cancer

Early studies examining the use of chest radiographs to screen for lung cancer demonstrated unacceptably low sensitivity, such as in the National Lung Screening Trial (NLST) looking at screening via computed tomography (CT) scans in the high risk population. This study demonstrated that high risk individuals undergoing screening via CT scans have a 20% reduction in lung cancer related mortality [6]. This has led to the American Cancer Society’s guidelines now recommending low dose CT scans in high risk patients (individuals between 55 and 74 years of age, who have a 30-year pack smoking history and currently smoke or have quit in the past 15 years) [6]. However, CT scans have a low specificity of only 61% when screening for lung cancer. For approximately every one true positive scan there are 19 false positive scans [7]. This is problematic in two major ways: it results in anxiety and fear for the patient and their family, and it can result in unnecessary follow-up interventions including invasive biopsies or surgical resection. Finally, while fluorodeoxyglucose-positive emission tomography (FDG-PET) scans have been shown to have a specificity and sensitivity of nearly 90% in the high risk patient, their utility as a screening test cannot currently be supported due to issues surrounding high cost and poor availability [3]. Unfortunately, the only definitive method for diagnosing lung cancer is biopsies.

Therefore, there is a vital need for development of minimally-invasive, cost-effective, easy to administer and approachable tests that can replace or be used in conjunction with CT scans to augment the low specificity value scans have on their own. We believe the answer to this problem is through investigation of molecular biomarkers in circulating samples and new non-invasively collected bodily fluids or aerosols such as exhaled breath, sputum, and urine [3,4]. These samples are easily obtainable and can be analyzed for molecular biomarkers such as microRNAs (miRNAs), free DNA, gene methylated products and others [4]. Recently, miRNAs have been observed in bodily fluids and are proposed as stable and reproducible markers [2,4,8]. Evidence of miRNA’s role in lung cancer was first seen by Volinia *et al.* [9] and subsequent studies have explored and solidified the diverse role that miRNAs play in the process of oncogenesis ranging from cell cycle regulation, apoptotic pathways, tumorigenesis and metastasis [2,3,4,5,8,9,10].

## 3. Brief Introduction to miRNAs

miRNAs are endogenous 19–22 nucleotide-long non-coding sections of RNA molecules that are involved in regulating the expression of target genes. They are involved in post-transcriptional gene silencing by RNA degradation or by inhibiting initiation of translation [2,4,8]. These regulations can affect multiple genes and can also cause a single miRNA to regulate various steps in a molecular pathway. Crucially, many studies have demonstrated that miRNAs are easily detectable in blood and are released in three different mechanisms: energy-free passive leakage from lysed cells, active release through microvesicles and active secretion in microvesicle free form [4,8]. Another mechanism being investigated is the release of intracellular miRNAs from exosomes into the extracellular space. Exosomes are small membrane vesicles inside the cell that fuse with multivescular bodies in the plasma membrane and are further released out into the extracellular environment [11]. The investigation of exosomal miRNAs is novel, but is also important as past research has shown that exosomes communicate between tumor cells and normal cells [12]. It has also been reported that tumor cells release higher concentrations of exosomal miRNA than normal cells [13]. Additionally, miRNAs show high stability in different types of biological samples. This is due to their resistance to endogenous and exogenous RNAses, extreme temperature and pH conditions, and repeated freeze-thaw cycles [1,4,8,14].

In fact, miRNA dysregulation has been reported under many different pathological and physiological conditions. Moreover, alterations of miRNA expression have been seen in different types of cancers such as hepatocellular carcinoma, lung, breast, gastric, pancreatic and many others [4,8]. Many studies have also shown higher circulating miRNA levels in cancer patients when compared to healthy individuals [4]. Additionally, many miRNAs show increased or decreased expression in blood and tumor tissue of cancer patients when compared with control patients; this differs from different tumor types [2,4,14].

In this review, current knowledge is described and potential future roles for single or panels of miRNA in early lung cancer detection are proposed. miRNAs are showing promise as biomarkers for lung cancer as they are altered in pathological stages and can be detected in biological fluids. Hence, if miRNA panels in high risk patients are shown to have both high sensitivity and specificity, they could be used as a widely applicable screening test as well as a minimally-invasive diagnostic test.

## 4. Literature Search and Study Selection

Studies were identified on the PubMed database using the search terms “miRNA” and “lung cancer” and either “sputum”, “whole blood”, “plasma” or “serum”. Initially identified studies were divided into sputum or circulating fluid. “Circulating fluid” indicates the studies using whole blood, plasma or serum as the biological fluid. The references list of eligible studies from the database was also manually searched, and included both basic scientific studies and clinical translation studies. No restrictions were applied to the years of searched literature because the use of miRNA in diagnosing lung cancer is a recent research task. As well, no explicit language restrictions were applied, but only English-language publications focusing on human studies were included in this review.

Identified studies were included in this review, provided that: (a) the study utilized one or many miRNAs and evaluated their role in usage for possible lung cancer screening and/or diagnosis; (b) the lung cancer diagnosis was confirmed through either histopathology or cytology; (c) sufficient data was available in order to accurately calculate clear specificity and sensitivity values; and (d) only research studies singularly and explicitly investigating lung cancer were included in this review.

Conference abstracts were excluded because of the limited data provided. Additionally, in studies where a training cohort and a validation cohort were available (mentioned in the results table), only the validation cohort was included in the results. No further statistical analyses were performed in this review. Two authors (J.G. and S.S.) independently reviewed all identified abstracts for inclusion in this review.

## 5. Analysis of Sputum and miRNA Profiling

The set of nine sputum studies range between the years 2010 to 2015, which are summarized in Table 1. Seven studies include data from the USA [4,15,16,17,18,19,20] and two studies include data from Canada [21,22]. The number of lung cancer patients enrolled per study range from 21 to 67 and the number of control patients enrolled range from 6 to 73. Diagnoses were confirmed through histopathology in eight studies [4,15,17,18,19,20,21,22], and cytology in one study [16]. In addition, four studies utilized a training/validation cohort [4,19,21,22]. miRNA assays were done by using either reverse transcription polymerase chain reaction (RT-PCR), quantitative real time polymerase chain reaction (qRT-PCR) or both.

miRNA profile reports included miRNA-21, 31, 126, 139, 143, 155, 200b, 205, 210, 372, 375, 429, 486, 708 and different internal controls. The sensitivity of using different miRNAs in sputum ranges from 61.5% to 100%, while the specificity ranges from 80% to 100% [4,9,16,17,18,19,20,21,22].

## 6. Analysis of Circulating Fluids; Whole Blood, Serum, and Plasma

The final set of 23 circulating fluid studies include blood (Table 2), serum (Table 3) and plasma (Table 4) investigation, ranging from 2011 to 2015. Twelve include data from China [23,24,25,26,27,28,29,30,31,32,33,34], five studies include data from the USA [35,36,37,38,39], three studies include data from Italy [40,41,42], one study includes data from both China and USA [43], one study includes data from France [44], and one study includes data from Poland [45]. The number of lung cancer patients enrolled ranges from 20 to 252 and the number of control patients enrolled ranges from 10 to 870. Diagnoses were confirmed through histopathology in nineteen studies [23,24,25,26,28,29,30,31,32,33,35,36,38,39,40,41,43,44,45] and cytology in one study [34], while cytology was used in three studies in conjunction with histopathology [27,37,42]. Additionally, eleven studies used a training cohort and literature to classify their miRNAs [25,26,27,31,33,35,36,37,38,41] while the other studies tested specific miRNAs based on literature searches [23,24,28,29,30,31,32,34,39,40,42,44,45]. miRNA assays were done by using either RT-PCR, qRT-PCR or both.

The miRNA profile reports are extensive and include mir-7, 10b, 15b, 20a, 21, 24, 25, 27b, 30d, 125a-5p, 125b, 126, 145, 152, 155, 182, 190b, 193a-3p, 194, 197, 199a-5p, 205, 210, 214, 221, 222, 223, 320, 328, 361-3p, 483-5p, 486-5p, 574-5p, 625, 652, 630, 660, 942, 944, 1254, 1284, 3662 and different internal controls. Studies by Sozzi *et al.* [35] and Sanfiorenzo *et al.* [44] did not report a specific miRNA but looked at large panels of miRNA. The sensitivity of using different miRNAs in circulating fluid ranges from 67% to 100%, while the specificity ranges from 66.4% to 100% [23,24,25,26,27,28,29,30,31,32,33,34,35,36,37,38,39,40,41,42,43,44,45].

## 7. Analysis of miRNA Studies

Research examining the role of miRNAs in lung cancer is a relatively new area of interest. The utility of miRNAs to serve as biomarkers for detection and diagnosis of lung cancer holds very promising potential for several reasons. Firstly, circulating miRNAs are found to be stable in the bloodstream and other biological fluids and are detectable in small quantities, making their use easy and cost-effective. Secondly, miRNA levels are altered in lung cancer patients when compared to healthy patients, making them potential markers of disease. In fact, miRNA panels could be used in synergy with current diagnostic methods and serve as excellent diagnostic, prognostic and predictive biomarkers.

In this paper, we have reviewed current literature reporting on the use of miRNAs as diagnostic biomarkers in lung cancer. The results suggest that a wide range of miRNAs could be used to possibly diagnose cancer using sputum as the biological sample with a sensitivity range of 61.5% to 100% and a specificity of 80% to 100%. When circulating blood fluids are used as the biological sample, lung cancer patients could be differentiated with a sensitivity range of 67% to 100% and a specificity of 66.4% to 100%. These results support the concept of using miRNAs as biomarkers for diagnosing cancers as they can discriminate between lung cancer (NSCLC or SCLC) patients and healthy controls. Additionally, in most of these studies, initial miRNAs were profiled using arrays and the most promising miRNAs were further validated using qRT-PCR or RT-PCR. Most studies identified a panel of miRNAs rather than one single miRNA, thus increasing diagnostic accuracy in discriminating between patients with lung cancer and healthy controls.

When considering studies that have shown miRNAs that are dysregulated in cancer patients compared to controls, it is important to consider the methodology used when interpreting the results. According to Baker *et al.* [46], it order to obtain proper statistical significance and power, studies should contain at least 70 cases and 110 controls. It is also stated that interpretation should be based on true positive and false positive values. True positive values are calculated as sensitivity (%), the probability that the biomarkers are positive in cancer cases. False positive values are calculated as specificity (%), the probability that the biomarkers are positive in the non-cancerous controls [46]. Although high sensitivities and specificities are desired, there is not a proper “cutoff” for what values constitute a proper screening/diagnostic test. Thus, for this review, when comparing sensitivity and specificity values between studies, we will define 80% or higher as a good screening/diagnostic test.

## 8. Sputum and miRNA Profiling

Various miRNAs were identified as potential biomarkers in sputum. The most commonly reported miRNA was miRNA-210, which was reported in seven studies. miRNA-21 was reported in five different studies, miRNA-31 was reported in four different studies, and miRNA-155 was reported in three studies. No study has used all four of these miRNAs in one panel. However, Roa *et al.* [21] and Kim *et al.* [22] reported a panel using miRNAs-21, 155, 210, 143, and 372 in 2013 and 2015, respectively. Roa *et al.* [21] presented a sensitivity of 100% and a specificity of 83.3% when discriminating between healthy patients and lung cancer patients. The Kim *et al.* [22] study resulted in a sensitivity of 67.8% and a specificity of 90% with a five miRNA panel.

In 2010, Yu *et al.* [19] were the first to identify a miRNA panel containing 4 different miRNAs including miRNA-21. Their panel differentiated healthy patients from patients with ADC with a sensitivity of 70.3% and a specificity of 80%. The same group again used a panel of 3 different miRNAs, including miRNA-210, to differentiate between healthy patients and patients with SCC. In the same study, Xing *et al.* [18] reported miRNAs-126, 139 and 429 as under expressed compared to controls, resulting in a sensitivity and specificity of 72% and 95%, respectively. In 2014, the same group utilized miRNA panels with miRNA-31 and 210, to differentiate between healthy patients and patients with ADC or SCC. This same study also evaluated the synergistic use of miRNA panels with CT-scans. In fact, combining both CT and miRNA panels provides a higher specificity of 91.2% (compared to 83.8%) and a similar sensitivity of 92.4% (compared to 93.9%) [4]. Additionally, three studies have used just two miRNAs (31 and 210) as possible discriminators with sensitivities ranging from approximately 62% to 66% and specificities ranging from 85% to 91% [4,15,17].

When comparing the studies investigating miRNA levels in sputum, it is important to interpret their results while taking into account their sample sizes, miRNA panel selection, and methodology. All of the studies used either RT-PCR or qRT-PCR to measure miRNA, allowing comparisons to be made between them. None of the studies had the recommended sample size of 70 cases and 110 controls, but Ulivi *et al.* [4] had the highest sample size with 64 cases and 73 controls. Using 80% as the cutoff for sensitivity and specificity, Roa *et al.* [21] presented a sensitivity of 100% and a specificity of 83.3% and Su *et al.* [20] presented a sensitivity of 83.7% and specificity of 87.5%. Of these 3 studies, Roa *et al.* [21] has the widest range of miRNA investigated, using a 5 miRNA panel, while the other 2 studies [4,20] used 2 or 3 miRNAs. Although these studies show promise, they do not have the proper sample size, thus more investigation is needed using sputum.

Although there are only nine studies that have evaluated miRNAs in sputum, it appears that sputum could be a promising biological sample given that it can be easily collected and analyzed. Furthermore, miRNA expression levels are extremely stable in sputum and do not change from day 1 to 7 post-collection, contributing to its potential as a viable biological sample to use for measuring miRNA [16]. Finally, the diagnostic yield of sputum could potentially be improved by utilizing early morning sputum samples or by obtaining multiple samples per patient.

## 9. Whole Blood and miRNA Profiling

Only four studies reported miRNA in whole blood samples with a sensitivity range of 70% to 88% and a specificity range of 76.9% to 100%. Due to the limited studies, all four studies reported different miRNAs profiles. Patnaik *et al.* [39] were the only ones to use a panel of four miRNAs while the other three studies used only one miRNA. All of the studies used either RT-PCR or qRT-PCR to measure miRNA, allowing comparisons to be made between them. None of the studies have the recommended sample size, but Yang *et al.* [34] had the highest with 74 cases and 52 controls. Two studies had sensitivities and specificities above the 80% cutoff, with Li *et al.* [32] having a sensitivity of 80% and specificity of 100%, and Patnaik *et al.* [39] have a sensitivity of 88% and specificity of 89%. With all studies using different miRNAs, there is much more exploration needed regarding measuring miRNA in whole blood when investigating lung cancer.

## 10. Serum and miRNA Profiling

miRNAs were also reported in isolated serum in a total of ten studies with sensitivities ranging from 69% to 100% and specificities ranging from 66.4% to 93.4%. The different miRNAs reported by all of these studies have no apparent explicit coherence. No common miRNA is seen in these studies, yet regardless, the reported values of sensitivity and specificity appear promising. Bianchi *et al.* [41] reported a 34 miRNA panel that discriminated healthy patients and patients with AC or SCC with a sensitivity of 71% and a specificity of 90%. Both Chen *et al.* [27] and Hennessey *et al.* [38] obtained very similar results. Hennessey *et al.* [38] selected a pair of miRNA-15b and 27b and obtained 100% sensitivity and 84% specificity after evaluating 328 different miRNAs. Jiang *et al.* [30] also reported a high sensitivity value of 96% and a high specificity value of 90% using only a singular miRNA-205. In 2015, Wang *et al.* [31] reported a sensitivity and specific of 87.5% using miRNA-125a-5p, miRNA-25, and miRNA-126. This same year, a study from the same group [33] investigated miRNA-194, 652, and 660 together in a population in China, resulting in a sensitivity of 85.9% and a specificity of 93.4%. They also collaborated with cohorts in the USA while looking at miRNA-483-5p, 193a-3p, 214, 25, and 7 with a sensitivity and specificity of 89% and 68%, respectively [43].

All studies used either RT-PCR or qRT-PCR, allowing comparisons to be made between them. Five studies had high sample sizes, ranging from 142 to 252 cases and 110 to 217 controls [27,28,31,33,43] but only three of these studies were above the sensitivity and specificity cut off, with sensitivities ranging from 85.9% to 92.5% and specificities ranging from 82.6% to 93% [27,31,33]. Despite this, there was not coherence in which miRNAs are most suitable when investigating lung cancer in serum, thus there is more work needed to determine which miRNAs are mist important when detecting lung cancer.

## 11. Plasma and miRNA Profiling

Similarly, various miRNA were investigated in the nine studies using plasma isolated from whole blood as a biological sample, with sensitivities ranging from 67% to 91.7% and specificities ranging from 68% to 96.6%. miRNA-21 was reported in five studies and miRNA-155, 210 and 486-5p was all reported in two total independent studies. Shen *et al.* [35,36], wrote two papers in 2011 using miRNA panels of miRNA-21, 210 and 486-5p in one paper and adding miRNA-126 to the second paper; they reported sensitivity of 76.3% and 86.2% and specificity of 85% and 97%, respectively. Geng *et al.* [26] investigated five miRNAs using qRT-PCR techniques. miRNA-20a, miRNA-223, miRNA-21, miRNA-221, and miRNA-145 were found to be significantly altered when comparing healthy controls and NSCLC patients. However, these miRNA were all investigated separately rather than in a panel. They reported sensitivities ranging 70%–87% and specificities ranging 68%–87%. There was also a significant difference between the NSCLC patients and the non-cancerous pulmonary disease controls, reporting a sensitivity ranging 67%–87% and a specificity ranging 68%–86%. However, a difference was not seen in four of the five miRNA when comparing the healthy controls and the non-cancerous pulmonary disease controls, having a sensitivity range of 52%–72% and a specificity range of 50%–75%. Conversely, Sanfiorenzo *et al.* [46] reported statistical miRNA differences in patients with chronic obstructive pulmonary disease (COPD) in comparison and conjunction with healthy volunteers and lung cancer patients. Sanfioenzo *et al.* [46] had a very small sample size, and did not look at all of the same miRNA as Geng *et al.* [26]. Further studies with proper sample sizes are needed to determine if there is a difference between miRNA in NSCLC and COPD/non-cancerous pulmonary disease. In 2014, Sozzi *et al.* [18] have demonstrated the potential of using miRNA in screening programs where they used a miRNA signature classifier together with CT and reported sensitivity of 98% with a 3.7% false positive rate. Very recently, a group in Poland were the first to investigate miRNA-944, and 3662 in this setting. Through the use of qRT-PCR, Powrozek *et al.* [45] found a sensitivity and specificity of 91.7% and 85.7%, respectively, using the combined two miRNAs.

Out of these studies investigating miRNA in plasma, all used RT-PCR or qRT-PCR, but none had suitable sample sizes, with the highest being 126 cases and 102 controls [26].

All three samples isolated from circulating fluid show promising avenues of use in future diagnosis. In comparison with sputum, there are more studies evaluating the role of miRNAs in circulating fluid. However, there are few consensuses in the miRNA panel used in circulating fluid to discriminate between healthy controls and lung cancer patients when compared to sputum. Regardless, using miRNAs in circulating fluids appears to result in a greater total range of specificity and sensitivity when compared to sputum. This is likely due to the nature of how sputum is collected, as it is coughed up and can have a varying amount of cells from the patient. Overall, circulating fluids are more consistent than sputum, making them a better medium for measuring miRNA.

## 12. Conclusions and Future Directions

As this review demonstrates, miRNA-based screening and diagnosis have a long way to go before they can be applied in the clinical setting. There is some consensus in the miRNA used in sputum (miRNA-21, 31, 210, 155) but the miRNAs used in circulating fluid show little unified reporting. Interestingly, miRNA-21 is reported by five sputum studies and five plasma studies making it a useful biomarker start point. However, it is unlikely that any single miRNA will be used since most studies utilize panels of miRNAs as they increase the specificity and sensitivity of diagnosis.

When comparing the different mediums to measure miRNA, it seems that serum and plasma are the better alternatives compared to sputum and whole blood. Past research has shown issues with using whole blood to measure miRNA, as the red blood cells affect the miRNA levels. Sputum, however, is also problematic as samples taken from patients can have varying amount of cells in them. In addition, many patients have comorbidities such as COPD, making coughing up sputum a difficult task.

Using more than one miRNA is important when trying to detect lung cancer, but there is little consensus to which miRNA are best to use. miRNA-21, 31, 143, 145, 155, 210 and 372 are the most commonly investigated miRNAs thus far, therefore it would be beneficial to look at these 7 miRNAs in one panel, and to further narrow down the top contenders in subsequent studies with proper sample sizes.

An area that needs to be explored more is using miRNA in conjunction with CT scans. Xing *et al.* [18] showed that when combining CT scanning with miRNA measurement, the sensitivity value increased and the specificity value remained approximately the same. However, other studies have not investigated the beneficial effects of combining the two tests.

Xing *et al.* [18] reported miRNAs-126, 139 and 429 as under expressed compared to controls, resulting in a sensitivity and specificity of 72% and 95%, respectively. In 2014, the same group utilized miRNA panels with miRNA-31 and 210, to differentiate between healthy patients and patients with ADC or SCC. This same study also evaluated the synergistic use of miRNA panels with CT-scans. In fact, combining both CT and miRNA panels provides a higher specificity of 91.2% (compared to 83.8%) and a similar sensitivity of 92.4% (compared to 93.9%).

There is much interest and excitement that miRNAs could represent a significant advancement as they represent a quicker, cost-effective, less invasive and are possibly synergistic modality to current diagnostic methods. However, any biomarker based assay with hopes to have clinical applicability needs to demonstrate robustness and reproducibility. There needs to be extensive, systematic and cohesive future reporting done on miRNAs in both sputum and circulating fluid miRNA profiles, since currently there was very little overlap in the current profiles of miRNAs with varying sample sizes between studies. In fact, even the controls used were not similar in the studies for both sample streams. Moreover, consensus amongst research groups should be sought to allow standardization of protocols and reproducibility of results.

In terms of experimental methods, future work should focus on identifying panels of miRNAs that can provide optimal diagnostic accuracy. Priority also needs to be placed on identifying training cohorts before using a validation cohort. This will help in identifying a greater set of miRNA panel combinations and ensure rigorous research methodology and future consensus.

There is optimism and promise in the future use of miRNAs as non-invasive biomarkers for diagnosing lung cancer. However, a number of issues need to be addressed: (1) standardized protocol; (2) normalization of controls; (3) most advantageous biological sample(s); and, (4) optimal miRNA panels. Despite these hurdles, we believe that miRNAs represent the future as both a screening and non-invasive diagnostic test for lung cancer, allowing more patients to access potentially curative therapies.

## Figures and Tables

**Table 1:** Summary of sputum miRNA information in lung cancer.

Reference	Year	Country	Cases	Controls	Comparative Test	Discovery Phase
[19]	2010	USA	64	58	Histopathology	Yes
[18]	2010	USA	67	55	Histopathology	Yes
[16]	2010	USA	23	17	Cytology	Yes
[21]	2012	Canada	24	6	Histopathology	Yes
[15]	2013	USA	39	42	Histopathology	Yes
[4]	2014	USA	64	73	Histopathology	Yes
[17]	2014	USA	35	40	Histopathology	Yes
[22]	2015	Canada	21	10	Histopathology	Yes
[20]	2015	USA	56	73	Histopathology	Yes
**Reference**	**Methods**	**miRNA Assay**	**miRNA Profiling**	**Sensitivity (%)**	**Specificity (%)**	
[19]	Literature and training set (36 cases and 36 controls)	RT-PCR and qRT-PCR	miRNA-21 ,200b, 375, 486	70.3	80	
[18]	Literature and training set (48 cases and 48 controls)	RT-PCR	miRNA-205, 210, 708	72	95	
[16]	Literature	RT-PCR	miRNA-21, 155	69.6	100	
[21]	Literature and training set (4 cases and 4 controls)	RT-PCR and qRT-PCR	miRNA-21, 143, 155, 210, 372	100	83.3	
[15]	Literature	qRT-PCR	miRNA-31, 210	61.5	90.5	
[4]	Literature and training set (66 cases and 68 controls)	qRT-PCR	miRNA-31, 210	64.1	89.2	
[17]	Literature	qRT-PCR	miRNA-31, 210	65.7	85	
[22]	Literature	RT-PCR and qRT-PCR	miRNA-21, 143, 155, 210, 372	67.8	90	
[20]	Literature	qRT-PCR	miRNA-21, 31, 210	83.7	87.5	

**Table 2:** Summary of whole blood miRNA information in lung cancer.

Reference	Year	Country	Cases	Controls	Comparative Test	Discovery Phase
[32]	2011	China	20	10	Histopathology	Yes
[39]	2012	USA	22	23	Histopathology	Yes
[42]	2013	Italy	86	24	Histopathology/Cytology	Yes
[34]	2015	China	74	52	Cytology	Yes
**Reference**	**Methods**	**miRNA Assay**	**miRNA Profiling**	**Sensitivity (%)**	**Specificity (%)**	
[32]	Literature	RT-PCR	miRNA-21	80	100	
[39]	Literature	qRT-PCR	miRNA-190b, 630, 942, 1284	88	89	
[42]	Literature	RT-PCR	miRNA-328	70	89	
[34]	Literature	qRT-PCR	miRNA-10b	86.5	76.9	

**Table 3:** Summary of serum miRNA information in lung cancer.

Reference	Year	Country	Cases	Controls	Comparative Test	Discovery Phase
[41]	2011	Italy	34	30	Histopathology	Yes
[37]	2011	USA	22	31	Histopathology/Cytology	Yes
[27]	2012	China	200	110	Histopathology/Cytology	Yes
[38]	2012	USA	55	75	Histopathology	Yes
[28]	2012	China	193	110	Histopathology	Yes
[29]	2013	China	60	30	Histopathology	Yes
[30]	2013	China	20	20	Histopathology	Yes
[31]	2015	China	142	111	Histopathology	Yes
[33]	2015	China	252	144	Histopathology	Yes
[43]	2015	China & USA	221	217	Histopathology	Yes
**Reference**	**Methods**	**miRNA Assay**	**miRNA Profiling**	**Sensitivity (%)**	**Specificity (%)**	
[41]	Literature and training set (25 cases, 39 controls)	qRT-PCR	34 miRNA panel	71	90	
[37]	Literature and training set (11 cases, 11 controls)	qRT-PCR	miRNA-1254, 574-5p	73	71	
[27]	Literature and training set	qRT-PCR	miRNA-20a, 24, 25, 145, 152, 199a-5p, 221, 222, 223, 320	92.5	90	
[38]	Literature and training set	qRT-PCR	miRNA-15b, 27b	100	84	
[28]	Literature	qRT-PCR	miRNA-125b	78.2	66.4	
[29]	Literature	RT-PCR	miRNA-210	69	87.2	
[30]	Literature	qRT-PCR	miRNA-205	96	90	
[31]	Literature and training set (24 cases, 24 controls)	qRT-PCR	miRNA-125a-5p, 25,126	88	82.6	
[33]	Literature and training set (44 cases, 22 controls)	qRT-PCR	miRNA-194, 652, 660	85.9	93.4	
[43]	Literature and training set (31 cases, 31 controls)	qRT-PCR	miRNA-483-5p, 193a-3p, 214, 25, 7	89	68	

**Table 4:** Summary of plasma miRNA information in lung cancer.

Reference	Year	Country	Cases	Controls	Comparative Test	Discovery Phase
[35]	2011	USA	76	80	Histopathology	Yes
[36]	2011	USA	58	29	Histopathology	Yes
[23]	2011	China	74	68	Histopathology	Yes
[24]	2011	China	63	30	Histopathology	Yes
[44]	2013	France	52	20	Histopathology	Yes
[25]	2013	China	34	32	Histopathology	Yes
[40]	2014	Italy	69	870	Histopathology	Yes
[26]	2014	China	126	102	Histopathology	Yes
[45]	2015	Poland	90	85	Histopathology	Yes
**Reference**	**Methods**	**miRNA Assay**	**miRNA Profiling**	**Sensitivity (%)**	**Specificity (%)**	
[35]	Literature and training set	qRT-PCR	miRNA-21 ,210, 486-5p	76.3	85	
[36]	Literature and training set	qRT-PCR	miRNA-21, 126, 210, 486-5p	86.2	96.6	
[23]	Literature	qRT-PCR	miRNA-155, 182, 197	81.3	86.8	
[24]	Literature	qRT-PCR	miRNA-21	76.2	70	
[44]	Literature	qRT-PCR	11 miRNAs	81.1	82.9	
[25]	Literature and training set (62 cases, 60 controls)	qRT-PCR	miRNA-21, 145, 155	76.5	81.3	
[40]	Literature	RT-PCR	miRNA signature classifier	87	81	
[26]	Literature and training set (25 cases, 25 controls)	qRT-PCR	miRNA-20a, 223, 21, 221, 145 (looked at separately)	83, 78, 67, 86, 70, respectively	81, 86, 68, 84, 68, respectively	
[45]	Literature	qRT-PCR	miRNA-944, 3662	91.7	85.7	
